# The Effect of Judo Training on Set-Shifting in School Children

**DOI:** 10.1155/2019/2572016

**Published:** 2019-01-21

**Authors:** Wai Leung Ambrose Lo, Zhenwen Liang, Wenfeng Li, Suying Luo, Zhi Zou, Shaozhen Chen, Qiuhua Yu

**Affiliations:** ^1^Department of Rehabilitation Medicine, The First Affiliated Hospital, Sun Yat-sen University, Guangzhou, China; ^2^Guangdong Engineering and Technology Research Centre for Rehabilitation Medicine and Clinical Translation, Department of Rehabilitation Medicine, The First Affiliated Hospital, Sun Yat-sen University, Guangzhou, China; ^3^Department of Rehabilitation Medicine, The First Affiliated Hospital of Jinan University, Guangzhou, China; ^4^Applied Cognitive Neuroscience Laboratory, Department of Rehabilitation Sciences, The Hong Kong Polytechnic University, Hong Kong

## Abstract

Improving executive functions (EFs) is desirable as they are considered to be critical for academic attainment and mental wellness in children. The aim of this study was to explore the effect of Judo training on the set-shifting function using a spatial task-switching paradigm. Protocol  1 compared the set-shifting ability of Judo players with age-matched healthy individuals. Protocol  2 compared the difference in EFs between children who underwent Judo training (intervention) and age-matched controls. EFs were assessed by a spatial task-switching test. Error rates and response times were analysed using two-way repeated-measures ANOVA.* Protocol 1. *The group effect on error rates was significant. The trial type × group effect was significant in the Judo group. Error rates in the Judo group were lower in the switch trials than the control group (*p *= 0.001). No significant group difference was seen in the repeat trials (*p *= 0.764).* Protocol* *2*. The time × trial type × group effect was significant. Post hoc analysis showed significantly lower error rates by the intervention group on switch trials compared to the control group (*p *= 0.006). Regular Judo training may potentially be an option for improving EFs in schoolchildren or in populations with executive dysfunction.

## 1. Introduction

Executive functions (EFs) refer to the ability to concentrate and think. The three core elements of EFs are inhibition, working memory, and mental set-shifting [1, 2]. EFs are considered to be critical for academic attainment and mental wellness in young children [3, 4]. Executive dysfunction in childhood often leads to EF deficits in adulthood [5] and does not disappear without intervention [6]. Improving EFs early in life is, therefore, desirable [7]. Set-shifting is a type of EF that refers to the flexibility to switch between different goals or switch between multiple actions rules [8]. Set-shifting may facilitate goal attainment by overcoming interference from alternative goals or means [9, 10]. Set-shifting is of importance to daily life since it enables juggling between different self-regulatory goals and the associated meanings of goals that can be attained, leading to successful self-regulation [2]. Traditional martial arts training is one of the strategies that has been demonstrated to have a positive impact on EFs in young children.

Martial arts are a type of open-skilled sports that requires players to react in a dynamically changing and externally paced environment [11, 12]. Previous studies have provided empirical evidence to support that regular participation in martial arts, such as Taekwondo [13, 14] and mixed martial arts [15], could facilitate improvement in EFs. Cho et al. (2017) found that the children who underwent 16 weeks of Taekwondo training had better performance in the Stroop Color and Word Test than those without Taekwondo training. The intense level of cognitive demands (e.g., planning, problem-solving, and set-shifting) and physical demands (e.g., cardio fitness, strength and endurance, and movement coordination) may play a role in facilitating EF development by stimulating neuroplasticity-related growth factors such as brain-derived neurotrophic factor (BDNF) [13, 16]. However, previous studies focused on the effects of martial arts on inhibition, which may not be generalized to set-shifting.

Judo is a type of martial art that primarily involves grappling and throwing. Based on the existing literature, the injury rate of Judo is lower than other forms of martial arts, making it an increasingly popular martial art practice among school children [17]. The injury rate for Judo players is approximately 12% [18] as opposed to 59% in Taekwondo, 51% in Aikido, 38% in Kung Fu, and 30% in Karate [19]. To date, there are very limited studies that investigate the effect of Judo training on EFs in young children. It is important to determine whether Judo training has a positive impact on EFs before it can be recommended as a mean to improve set-shifting function.

This study aimed to explore the effect of Judo training on the ability to set-shift using a spatial task-switching test that required participants to switch between left-right and up-down spatial dimensions. Two protocols were conducted in the present study. The first protocol investigated the modulation of long-term Judo training on set-shifting function. The second protocol investigated the causal relationship between Judo training and set-shifting. We hypothesized the following: (1) Participants with long-term Judo training would perform better on the spatial task-switching test than those without long-term Judo training. (2) The performance on spatial task-switching would be significantly improved in participants who received eight weeks of Judo training compared to age-matched controls.

## 2. Materials and Methods

### 2.1. Experimental Design

**Figure 1:**
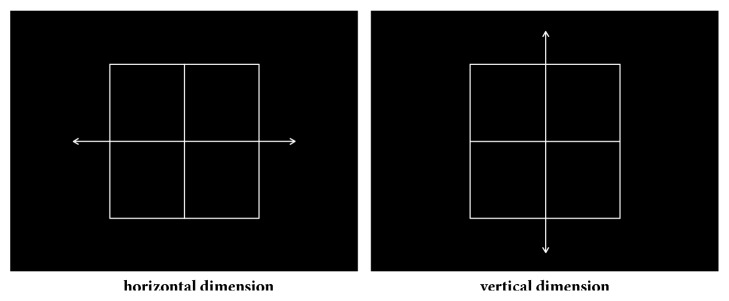
The two spatial dimensions in the spatial task-switching test.

**Figure 2:**
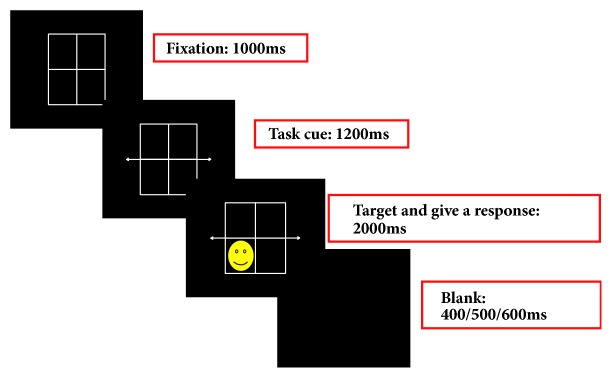
Schematic illustration of one typical trial in the spatial task-switching test.

Protocols 1 and 2 employed the same spatial task-switching test to assess set-shifting ability. Participants were shown a grid that was divided into four squares. In the centre of the grid was a double-headed arrow pointing either horizontally or vertically. A smiley face randomly appeared in any one of the four squares. Participants were asked to shift their attention and response between the horizontal and vertical dimension (two spatial dimensions). The horizontal dimension was relevant when a horizontal double-headed arrow divided the four squares into left and right panels. The vertical dimension displayed as the vertical double-headed arrow divided the four squares into high and low panels.  Figure 1 shows the configuration of the two spatial dimensions of the spatial task-switching test. The time course of a typical trial in the spatial task-switching test is illustrated in Figure 2. At the beginning of a trial, a grid as the fixation appeared on the screen for 1000 ms. This was followed by a task cue that lasted for 1200 ms. The task cue was a double-headed arrow pointing vertically or horizontally in the four squares. The target stimulus, a smiley face, then appeared for 2000 ms, along with a double-headed arrow pointing vertically or horizontally. When the target stimulus appeared, participants were asked to make their responses as soon as they could. Once the response was completed or 2000 ms passed, a blank screen appeared for 400, 500, or 600 ms. The participants were asked to press the “v” key on the keyboard if the smiley face appeared in the lower panel of the vertical dimension or the left panel of the horizontal dimension. If the smiley face appeared in the upper panel of the vertical dimension or the right panel of the horizontal dimension, participants were asked to respond by pressing the “h” key on the keyboard. On repeat trials, the spatial dimension was the same as that on the previous trial. On switch trials, the spatial dimension was different from the previous trial. The ratio of repeat trials to switch trials was 1:1. The location of the smiley face and sequence of repeat or switch trials were pseudorandom. There were 40 trials in each block and two blocks in total. The time for completing the spatial task-switching test was around 25 minutes.

### 2.2. Ethical Considerations

Ethical approval of the entire study was obtained from the ethical committee of the Hong Kong Polytechnic University. A participant information sheet was provided to all participants. The study procedure and possible risks were explained to all participants and their parents by a member of the research team. Participants were informed that they could withdraw from the study at any time without giving a reason. Informed consent was obtained from all participants and their parents. All procedures were performed in accordance with the Declaration of Helsinki.

### 2.3. Protocol 1

#### 2.3.1. Sample Population

Judo players and controls were recruited from a secondary school in Hong Kong. The inclusion criteria for Protocol 1 were as follows: (1) aged between 12 to 16 years; (2) healthy or corrected-to-healthy visual acuity. Additional inclusion criteria for only the Judo group were a minimum of four years of Judo training and participating in at least three sessions of Judo training per week. Age-matched, healthy school children who had never participated in any form of martial arts were recruited as controls. Exclusion criteria for Protocol 1 were as follows: (1) history of a neurological disorder or mental disorder or being on regular medication; (2) presence of acute injury during the data collection period; (3) participation in any other martial arts training.

#### 2.3.2. Procedure

Participants who agreed to take part in the study were invited to attend a single data recording session. Each participant completed a demographic information sheet that collected personal information, time spent in formal Judo training per week, and regular participation in extracurricular activities (e.g., piano) and other sports. Each participant was asked to sit comfortably on a chair in front of a desktop computer with an upright and relaxed posture. A 15-inch CRT monitor for displaying visual stimuli was placed at a distance of 65-75 cm away from participant's vision. All of the participants were provided with 30 practice trials. Participants were required to demonstrate an accuracy rate of 90% on these 30 trials before taking part in the data recording session. The spatial task-switching test was presented using E-prime 2.0 software (Psychology Software Tools, Inc., Pittsburgh, PA, USA). Completion of the whole experiment took approximately 35 minutes.

#### 2.3.3. Data Analysis

Statistical analyses were performed using SPSS v20.0 IBM; SPSS Inc. Chicago, IL, USA. Sample population characteristics of the two groups were analysed by descriptive analysis. The between-group differences in demographic variables were tested using the independent t-test. Behavioural data of the task-switching paradigm, including error rates and response times, were analysed using two-way repeated-measures ANOVA. The within-subject factor was the trial type (repeat or analyses) and the between-subject factor was the group (Judo training or control group). Weight and height were included as the covariates in the one-way ANCOVA as weight (*t* = 2.19,* p* = 0.038) and height (*t* = 2.11,* p* = 0.046) significantly differed between the Judo training and control groups. Age was included as a covariate as the Judo group had a higher mean age than the control group. Post hoc pairwise comparisons with Bonferroni adjustment were applied when significant interaction effects were observed. The Greenhouse-Geisser correction was used when Mauchly's test of sphericity was violated. The significance level was set at* p *< 0.050 for the whole model. Partial eta-squared (*η*^2^_p_) was reported to demonstrate the effect size in the two-way repeated-measures ANOVA tests.

### 2.4. Protocol 2

#### 2.4.1. Sample Population

The participants in Protocol 2 were recruited from a secondary school in Hong Kong. The inclusion criteria for Protocol 2 were as follows: (1) Aged between 12 to 16 years; (2) never having received Judo training; (3) healthy or corrected-to-healthy visual acuity. Exclusion criteria for Protocol 2 were as follows: (1) history of neurological disorder or mental disorder or being on regular mediation; (2) presence of acute injuries during the data collection period. Participants had a choice to either participate in Judo training or not. Those who would like to participate in Judo training were included in the intervention group. The participants in the control group were recruited by stratified sampling in different year groups of the school.

#### 2.4.2. Intervention

Participants in the intervention group received Judo training three times a week for eight weeks. Each training session consisted of 5 minutes of warming up, 20 minutes of physical fitness training, 60 minutes of Judo practice, and 5 minutes of cooling down. Physical fitness training included shuttle run, speed training, and strength exercises. The Judo practice session included 20 minutes of falling techniques (throw down, push down, and hook down) and 40 minutes of Randori (20 minutes of Judo fighting on the ground and 20 minutes of standing Judo fighting). The attendance rate for the eight-week Judo program was 100%.

#### 2.4.3. Procedure

Each participant completed the same demographic data collection procedure as described in Protocol 1. All of the participants received the spatial task-switching test at baseline and after the intervention. The same paradigm of spatial task-switching described in Protocol 1 was employed.

#### 2.4.4. Data Analysis

The differences in demographic variables between the groups (experimental and control groups) were tested using an independent t-test. The effects of Judo training on error rates and response time of the spatial task-switching test were analysed by three-way repeated-measures ANOVA: the within-subject factors were time (baseline or postintervention) and trial type (repeat or switch); the between-subject factor was the group (experimental or control). The between-group differences in the change of error rates and response time between baseline and postintervention were investigated using a two-way repeated-measures ANOVA. The within-subject factor was the trial type (repeat or switch) and the between-subject factor was the group (experimental or control). Post hoc pairwise comparisons were applied when a significant interaction effect was observed. The Greenhouse-Geisser correction was used when Mauchly's test of sphericity was violated. The significance level was set at* p *< 0.05. Partial eta-squared (*η*^2^_p_) was reported to demonstrate the effect size in the three-way repeated-measures ANOVA tests.

## 3. Results

### 3.1. Protocol 1

#### 3.1.1. Sample Characteristics

Twenty-six participants in total were recruited to take part in Protocol 1. Twelve Judo players (three females and 9 males) met the inclusion criteria for the Judo group. Fourteen age-matched participants (three females and 11 males) were recruited for the control group. All participants were right-handed and took part in the weekly physical education curriculum. None of the participants reported regular participation in extracurricular activities (e.g., piano) or other sports.  Table 1 describes the demographic characteristics of the sample populations.

#### 3.1.2. Spatial Task-Switching Test

**Figure 3:**
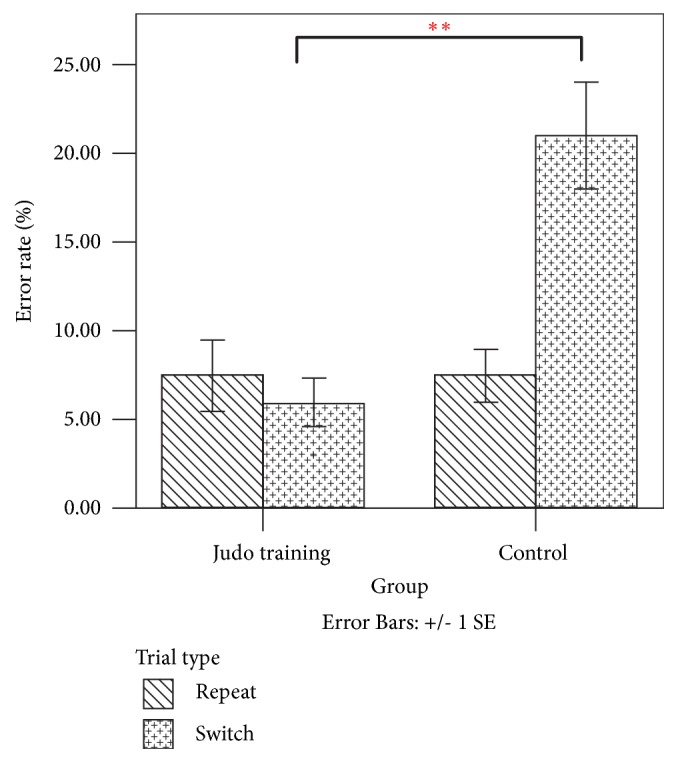
Error rates of repeat and switch trials in spatial task-switching test for Judo training and control groups. *∗∗ denotes that p < 0.01.*

By adjusting for age [*F *(1, 21) = 0.517,* p *= 0.480, *η*^2^_p_ = 0.024], weight [*F *(1, 21) = 0.006,* p *= 0.939, *η*^2^_p_ < 0.001], and height [*F *(1, 21) = 0.009,* p *= 0.926, *η*^2^_p_ < 0.001], the main effect of the trial type on error rates was not statistically significant [*F *(1, 21) = 0.550,* p *= 0.467, *η*^2^_p_ = 0.026]. However, the group effect (Judo training or control) on error rates was statistically significant [*F* (1, 21) = 9.077,* p* = 0.007, *η*^2^_p_ = 0.302].  Figure 3 presents a graphical illustration of the error rates from the repeat and switch trials in the spatial task-switching test between the Judo training and control groups. The trial type × group effect on error rates was statistically significant [*F *(1, 21) = 8.522,* p *= 0.008, *η*^2^_p_ = 0.289]. The post hoc analyses of the interaction effect showed no statistically significant difference between the Judo training and control groups in error rates during repeat trials (*p *= 0.764). The Judo training group demonstrated significantly lower error rates in the switch trials than the control group (*p *= 0.001).

**Figure 4:**
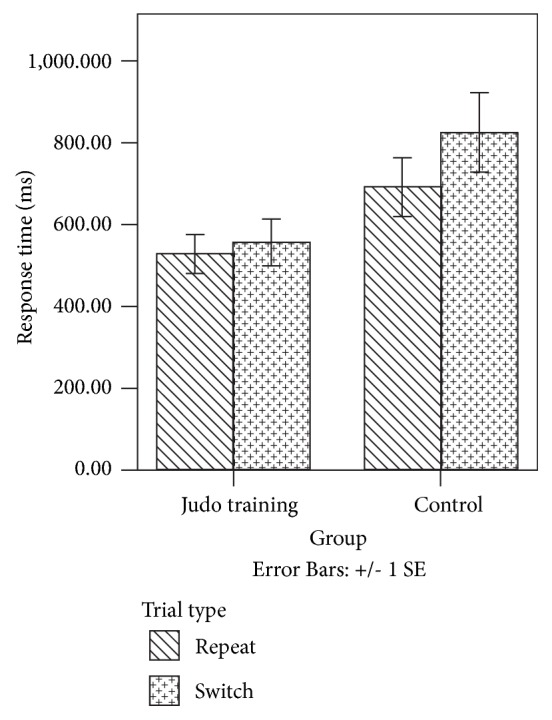
Response time of repeat and switch trials in spatial task-switching test for Judo training and control groups. ms: millisecond; SE: standard error.

After adjusting the age [*F *(1, 21) = 0.548,* p *= 0.467, *η*^2^_p_ = 0.025], weight [*F *(1, 21) = 0.265,* p *= 0.612, *η*^2^_p_ = 0.012], and height [*F *(1, 21) = 0.081,* p *= 0.778, *η*^2^_p_ = 0.004], the main effects of trial type and group on response time were not significant [*F *(1, 21) = 0.260,* p *= 0.616, *η*^2^_p_ = 0.012;* F *(1, 21) = 1.752,* p *= 0.200, *η*^2^_p_ = 0.077, resp.].  Figure 4 provides a graphical presentation of participants' response times during repeat and switch trials in the spatial task-switching test for the Judo training and control groups. The trial type × group effect on response time was not statistically significant [*F *(1, 21) = 2.294,* p *= 0.145, *η*^2^_p_ = 0.098].

### 3.2. Protocol 2

#### 3.2.1. Sample Population

Twenty-nine participants in total were recruited to take part in Protocol 2. Fourteen participants were recruited into the experimental group (three females and 11 males) and 15 age-matched participants (four females and 11 males) were recruited as controls. The sample population characteristics of Protocol 2 are presented in Table 2.

For error rates, the main effect of trial type (repeat or switch) was statistically significant [F (1, 27) = 82.691, p < 0.001, *η*2p = 0.754]. However, the main effects of time (at baseline or postintervention) [F (1, 27) = 1.826, p = 0.188, *η*2p = 0.063] and group (experimental or control) [F (1, 27) = 2.585, p = 0.120, *η*2p = 0.087] were not statistically significant (Table 3). The time × trial type × group effect on error rates was statistically significant [*F *(1, 27) = 10.631,* p *= 0.003, *η*^2^_p_ = 0.283]. Post hoc analysis on time × trial type × group effect was conducted separately at each time point. The trial type × group effect was only significant after Judo training [*F *(1, 27) = 20.077,* p *< 0.001, *η*^2^_p_ = 0.426] but not at baseline [*F *(1, 27) = 0.430,* p *= 0.518, *η*^2^_p_ = 0.016]. Hence, the group difference after Judo training was further examined at each trial type. After Judo training, both groups showed comparable error rates in repeat trials (*p *= 0.933). Participants in the intervention group showed significantly lower error rates in the switch trial than those in the control group (p = 0.006). These results indicated that participants in the Judo group had better posttest performance and more improvement on error rates in switch trials than the control group after eight weeks of intervention.

For the changes in error rates from baseline to postintervention, the main effect of trial type [*F *(1, 27) = 6.788,* p *= 0.015, *η*^2^_p_ = 0.201] and the trial type × group effect [*F *(1, 27) = 10.631,* p *= 0.003, *η*^2^_p_ = 0.283] were statistically significant. However, the group effect was not significant [*F *(1, 27) = 2.207,* p *= 0.149, *η*^2^_p_ = 0.076].

For response time, the main effects of time and trial type were statistically significant [F (1, 27) = 5.013, p = 0.034, *η*2p = 0.157; F (1, 27) = 31.778, p <0.001, *η*2p = 0.541, resp.]. However, the main effect of group and time × trial type × group effect were not significant, [F (1, 27) = 0.482, p = 0.493, *η*2p = 0.018; F (1, 27) = 1.033, p = 0.319, *η*2p = 0.037, resp.] (Table 4).

In terms of the changes in response time from baseline to postintervention, the main effect of trial type [F (1, 27) = 10.866, p = 0.003, *η*2p = 0.287] was statistically significant. However, the group effect [F (1, 27) = 1.156, p = 0.292, *η*2p = 0.041] and the trial type × group effect [F (1, 27) = 1.033, p = 0.319, *η*2p = 0.037] were not significant.

## 4. Discussion

The present study is among the first to investigate the effect of Judo training on school children's set-shifting ability. The first protocol indicated that school children with regular Judo training were better at set-shifting than controls. The second protocol indicated that set-shifting in school children may be enhanced by Judo training.

People participating in open-skilled sports, where they are required to anticipate in a constantly changing environment, are theorized to have better EFs than those participating in closed-skilled sports in a stable environment [11, 12, 20]. This theory is supported by data provided by Yu et al. (2017) in their investigation of the effect of open and closed skills on enhancing EFs in a cued task-switching paradigm. Yu et al. (2017) compared task switching in young adults who participated in open-skilled sports with those who participated in close-skilled sports. Results indicated that open-skilled sports participants performed better at task switching in a cued task-switching paradigm, as evidenced by their smaller difference in response time between switch and repeat trials than closed-skilled sports participants. This finding supports the results from Protocol 1 in the present study where significantly lower error rates on switch trials were observed in the Judo training group than the control group. Judo, as an open-skilled sport, requires the players to perform intense and complex motor actions in response to opponent's tactics to gain a competitive advantage in a changing environment [21]. Thus, Judokas are constantly required to anticipate their opponent's next action and prepare to switch from one action to another in an attempt to outmanoeuvre the opponent [11, 12]. The cognitive demands of Judo to respond to a constantly changing environment may enhance EFs [11, 22]. In addition, the specifics of Judo training, such as repetitive throwing and combat practice, are also known to improve aerobic capacity [21]. A systematic review by Best [23] suggested that aerobic physical activities that are also cognitively engaging are likely to have strong effects on EF processes.

The results from Protocol 2 provided further evidence to support the causal relationship between Judo training and set-shifting. This was evidenced by the comparable error rates during repeat trials for both groups but lower error rates on switch trial for those in the Judo group compared to the control group. The presence of larger improvements in error rates from baseline to postintervention for the Judo group compared to control group could further substantiate the effect of Judo training on EFs. The findings from Protocol 2 are consistent with those reported by Lakes & Hoyt [24]. Lakes & Hoyt assessed the cognitive and affective self-regulation ability of 207 school children who received three months of martial art training. Results indicated greater improvements in cognitive and affective self-regulation postintervention in the martial art training group compared to the control group, suggesting that participation in martial art training may have a beneficial effect on self-regulation. Despite the apparently positive results, the comparison of the findings between the two studies must be interpreted with caution. The present study provided Judo training in addition to the regular physical education curriculum whereas the study by Lakes et al. replaced two to three weekly sessions of physical education with martial art training. The findings of the present study indicated that it may be feasible to improve set-shifting function with Judo training. Cho et al. (2017) reported that the improvement in EFs by Taekwondo training was related to the increase in neuroplasticity-related growth factors, for example, BDNF [13]. Increased concentrations of BDNF and other neuronal growth factors are associated with significant improvements in cognitive functioning [25].

## 5. Limitations

The results of the present study should be interpreted with caution due to some limitations. One of the limitations was the nonrandomized allocation process for Protocol 2. This may introduce a bias towards the Judo group. The allocation procedure was used primarily for practical reason. The Judo programme was not funded by the study. It, therefore, seems unethical to impose a financial burden on those who did not wish to participate in Judo*. E*ven though the Judo group showed smaller error rates at the baseline compared to the control group in spite of trial type by visual inspection, the results of the changes in error rates between baseline and postintervention could further substantiate the effect of Judo training on EFs by teasing out the baseline group difference. The improvement seen in the Judo group was also higher than the control group. This should at least alleviate some of the concerns in terms of bias. Second, the sample size was not power calculated; thus it is likely to contain type II error. The small sample size increased SD in response time, which may lead to no significant main group effect and time × trial type × group effect on response time in Protocol 2. Accordingly, the interpretation of the changes in response time should be with caution. Third, the observed benefits of Judo on EFs may not be generalized to children who may have less interest in the sport. Future study is recommended to understand if such benefit could also be found in children who were not drawn to Judo. Fourth, the present study did not consider the confounding effects of IQ [26] and aerobic fitness [26] which may be related to EF. However, IQ had been shown to be more associated with inhibition than set-shifting [11, 12]. Future studies should tease out the confounding effects of these two factors. Fifth, participants in the control group did not receive additional training. It has been suggested that engaging in any new activity may produce benefits in EF. Thus, this study could not rule out that some of the improvement in EF observed in the Judo group may be related to participants engaging in a new activity. Future studies should consider incorporating a control intervention group to identify the relative contribution of Judo training to EF improvement.

## 6. Conclusions

This study offers evidence to support that participation in Judo training may be beneficial in improving set-shifting in school children. Regular Judo training may potentially be an option to improve EFs in school children or in populations where EFs are known to be affected such as attention deficit hyperactive disorder and autism spectrum disorder.

## Figures and Tables

**Table 1:** Demographic characteristics of the sample population.

	**Judo group** (**mean, SD**)	**Control group** **(mean**, **SD)**	***p*-value**
**Age**	14.25 (1.36)	13.43 (1.02)	*t* = 1.76, *p* = 0.091

**Weight (kg)**	50.67 (4.48)	46.14 (5.81)	*t* = 2.19, *p* = 0.038

**Height (cm)**	163 (4.57)	158.64 (5.77)	*t* = 2.11, *p* = 0.046

**BMI (kg/m** ^**2**^ **) **	19.04 (0.95)	18.26 (1.08)	*t* = 1.93, *p* = 0.065

Note: SD denotes standard deviation.

**Table 2:** Sample population characteristics for the intervention and control groups.

	**Experimental group** **(mean, SD)**	**Control group** **(mean, SD)**	***t- scores, p*-values**
**Age**	13.36 (1.15)	13.47 (1.24)	*t *= -0.245, *p *= 0.808

**Weight (kg)**	45.71 (6.73)	46.33 (5.92)	*t *= -0.263, *p *= 0.794

**Height (cm)**	158.00 (6.76)	159.53 (5.63)	*t *= -0.666, *p *= 0.511

**BMI (kg/m** ^**2**^ **) **	18.22 (1.44)	18.12 (1.27)	*t *= -0.115, *p *= 0.909

**Table 3:** Participants' error rates in repeat and switch trials stratified by group and time (error rate in %).

	**Intervention group**		**Control group**	
	Repeat	Switch	Repeat	Switch

**Baseline**	8.8±6.8	22.9±12.0	9.5±5.3	26.1±9.6

**Postintervention**	9.0±8.7	14.6±13.7	9.2±5.8	26.8±7.7

**Change**	-0.25±8.01	8.29±11.41	0.29±7.17	-0.67±7.15

**Table 4:** Participants' response times in repeat and switch trials stratified by group and time (response time in ms).

	**Intervention group**		**Control group**	
	Repeat	Switch	Repeat	Switch

**Baseline**	677.79±264.50	787.71±348.54	715.69±177.51	827.16±214.75

**Postintervention**	636.65±269.61	692.78±320.37	706.01±143.57	789.06±169.21

**Change**	41.14±138.67	94.93±146.16	9.67±85.60	38.11±80.01

## Data Availability

The data used to support the findings of this study are available from the corresponding author upon request.
